# Real-World Evidence of the Disease Burden and Economic Impact of Paroxysmal Nocturnal Hemoglobinuria in Italy

**DOI:** 10.3390/jcm14092889

**Published:** 2025-04-22

**Authors:** Roberta Bini, Lorena D’Anna, Diletta Valsecchi, Stefania Mazzoni, Valentina Perrone, Luca Degli Esposti

**Affiliations:** 1 Novartis Farma S.p.A., 20154 Milano, Italy; roberta.bini@novartis.com (R.B.); lorena.danna@novartis.com (L.D.); diletta.valsecchi@novartis.com (D.V.); 2 CliCon S.r.l. Società Benefit, Health, Economics & Outcomes Research, 40137 Bologna, Italy; stefania.mazzoni@clicon.it (S.M.); luca.degliesposti@clicon.it (L.D.E.)

**Keywords:** anti-complement therapy, C5/3-inhibitors, cost analysis, incidence, paroxysmal nocturnal hemoglobinuria, prevalence, real-world evidence

## Abstract

**Background/Objectives**: This analysis was conducted in Italy to estimate the epidemiology of paroxysmal nocturnal hemoglobinuria (PNH) and to describe the features and economic burden of PHN in the adult population considering the role of anti-complement therapy with C5/3-inhibitors (C5/3i). **Methods**: Administrative databases of healthcare entities covering approximately 12 million citizens were used to estimate the prevalence and incidence of PNH. Demographics, clinical characteristics and healthcare costs were analyzed among adults with PHN stratified by the presence/absence of C5/3i therapy. **Results**: The prevalence in Dec-2021 of PNH in adults was 17.6/1,000,000 people, and the incidence rate in the period 2011–2022 was 1.5/1,000,000/year. In 142 patients with at least 12 months of data available before and after inclusion (mean age: 50.7 years; 45.8% males), 27% received C5/3i therapy. The main baseline comorbidities were aplastic anemia and other bone marrow failure syndromes, found in 10.6% of patients and more common in C5/3i-treated than untreated patients (18.4% vs. 7.7%). Cost analysis showed that the average cost per patient per year (PPPY) was EUR 41,084, mainly driven by drug expenses (87% of total costs), especially anti-complement therapy (80%). RBC transfusions were the most impactive item among the hospitalization costs (EUR 1982 of EUR 4284 PPPY). The C5/3i-treated cohort was associated with higher total costs (EUR 133,472 vs. EUR 8089, *p* < 0.001), mainly due to drug expenses (EUR 127,180 vs. EUR 3217, *p* < 0.001). **Conclusions**: This real-world analysis confirmed a rising PNH prevalence in Italy, aligning with global data. Despite available therapies, many patients face a high disease burden, suggesting potential benefits from novel treatments targeting upstream complement components.

## 1. Introduction

Paroxysmal nocturnal hemoglobinuria (PNH) is a rare, acquired blood disorder caused by a somatic mutation in the hematopoietic stem cells of the gene encoding for phosphatidylinositol N-acetylglucosaminyltransferase subunit A (PIGA), causing the defective expression of glycosylphosphatidylinositol-anchored proteins (GPI-APs), including the complement regulatory proteins CD55 and CD59. The loss of CD55 and CD59 makes GPI-deficient red blood cells susceptible to complement-mediated hemolysis. PNH typically manifests with hemolytic anemia, bone marrow failure (BMF) and thrombosis [1,2].

The global incidence of PNH is estimated to be 1–1.5 cases per million people, although incidence rates might vary by region [3]. While earlier research suggested potential differences among ethnic groups, more recent findings indicate that the PNH incidence is relatively consistent across populations, though certain clinical characteristics may vary [4,5]. PNH presents similarly in both sexes, without familiarity, at any age, although the onset of the first symptoms is more common between the third and fourth decade of life [6]. There are also wide interindividual differences in clinical manifestations, including hemolytic anemia [2], hemoglobinuria [1], fatigue and smooth muscle dystonia [6,7,8].

If left untreated or treated only with supportive care, PNH can be a fatal disease; thrombophilia is the primary cause of mortality, accounting for 40–67% of deaths, distantly followed by kidney failure (8–18% of deaths) [9,10]. While in the past, patients with PNH generally lived for 10 to 22 years after diagnosis, the introduction of new drugs targeted at complement inhibition has brought great survival benefits, with most patients with PNH reaching a life expectancy nearly comparable to subjects without the condition [1].

Although there is no shared consensus on PNH classification yet, the International PNH Interest Group [11,12] has grouped the disease into three forms: (1) classical PNH, clinically characterized by evidence of IVH, with altered circulating serum levels of hemolysis biomarkers (increased LDH and fractionated bilirubin, decreased haptoglobin), or episodic hemoglobinuria in the absence of another BMF syndrome; (2) PNH in the setting of another BMF syndrome (i.e., aplastic anemia or myelodysplastic syndrome), with mild IVH; (3) subclinical PNH [11,12,13], without clinical or laboratory evidence of hemolysis or thrombosis and more commonly detected in patients with an underlying bone marrow abnormality. High-resolution flow cytometry can discriminate between the three disease phenotypes, which show a different clonal expansion of GPI-AP-deficient granulocytes, respectively, of large (above 50%), variable (below 50%) and small (below 10%) sizes [10,11,12,13]. This classification deserves particular attention for its repercussions for epidemiological estimates [3,14,15] as well as the therapeutic management of PNH [11,12]. For classical PNH, anti-complement therapy is the best frontline option, followed by an increase in dose/frequency, possibly combined with supportive care, or bone marrow transplantation in the case of an inadequate response. In the other two PNH forms, the main interventions should first focus on the coexisting BMF syndrome, but anti-complement therapy can be administered to patients with large PNH clones [11,12].

The latest pharmacological research is highly focused on anti-complement therapy aimed at inhibiting complement-mediated hemolysis [1,16,17]. However, agents targeting C5 (similar to eculizumab) are not fully curative, as some patients may still experience C3-mediated extravascular hemolysis and remain transfusion-dependent [16,18]. New agents able to intercept the initial activation steps of a complement cascade and prevent amplification downstream might represent a pioneering strategy to “shut down” hemolysis, thus providing a valuable tool for keeping PNH activity under control [19,20,21].

This analysis was carried out in a setting of real clinical practice in Italy to investigate the epidemiology, demographic characteristics, clinical status and therapeutic pathways of PNH in patients in Italy. The population was also assessed for disease progression and incidence of BMF. Lastly, healthcare resource consumptions and costs covered by the Italian National Health System (INHS) for the management of patients with PNH were analyzed.

## 2. Materials and Methods

### 2.1. Data Source

This observational retrospective analysis relied on integrated administrative data from a sample of Italian healthcare entities covering about 12 million residents, with data available from January 2010 to June 2023. The analysis used INHS-reimbursed healthcare resource databases, as previously described by our group [22]. Specifically, the following databases were used for the analysis: (1) a beneficiaries’ database to extract patients’ demographic data, namely, sex, age and date of death; (2) a pharmaceuticals database for all the information on medications reimbursed by the INHS, such as the Anatomical Therapeutic Chemical (ATC) code, the number of packages, the number of units per package, unit cost per package and prescription date; (3) a hospitalization database to collect hospitalization data, like discharge diagnosis codes classified according to the *International Classification of Diseases, Ninth Revision, Clinical Modification* (ICD-9-CM), the diagnosis-related group (DRG) and the DRG-related charge (supplied by the INHS); (4) an outpatient specialist services database to record data on specialist visits and diagnostic tests (date and type of provision, description of the activity, laboratory tests or specialist visit); (5) an exemption database to gather active payment waiver codes, by which patients are discharged from paying for services/treatments in the case of specific diagnoses.

The dataset used consists solely of anonymized data. Approval has been obtained from the ethics committees of the involved local health units. In line with Article 110 (processing of personal data for medical, biomedical or epidemiological research purposes) of the Italian Privacy Code, informed consent was waived, as obtaining it was deemed impossible or required a disproportionate effort.

### 2.2. Identification of the Study Population and Study Design

From January 2011 to December 2022 (inclusion period), all patients with PNH were identified by having had at least one hospitalization with a primary or secondary diagnosis of PNH (ICD-9-CM code 283.2) or an active exemption code for the pathology (RD0020).

The date of the first match with one of the above inclusion criteria was considered as the index date. All the patients were investigated for the whole period of available data before the index date. This characterization period of at least 12 months was used to assess previous treatments, comorbidities and clinical manifestations of PNH, hospital admissions, diagnostic procedures and outpatient specialist visits.

Patients were followed-up from the index date until the end of the available data (at least 6 months). During the follow-up period, drug prescriptions, comorbidities and clinical manifestations of PNH, hospitalization, diagnostic procedures and outpatient specialist visits were investigated. Moreover, healthcare resource use (HCRU) and the derived direct costs covered by the INHS were also analyzed.

### 2.3. Epidemiological Estimates of PNH

Epidemiological analyses of PNH were carried out for both the all-age population and the adult population only. Given that the hospitalization code and exemption code used to identify PNH does not allow discrimination between the three disease forms, epidemiolocal estimates were reported for the PNH population embracing all phenotypes.

The prevalence of diagnosed PNH, expressed as cases/1,000,000 people, was calculated as the number of prevalent patients alive on 31 December 2021 divided by the number of all health-assisted patients alive on 31 December 2021. Prevalence analyses were carried out on the entire period of available data. For each calendar year, the changes in prevalence proportions were evaluated using Poisson regression, estimating the prevalence proportion ratios (PPRs) with 95% confidence intervals (CIs).

The annual incidence of PNH in patients, expressed as cases/1,000,000 people, was calculated as the number of identified patients with PNH with an incident diagnosis each year from 2010 to 2022 divided by the number of person-years. The incident rates of each year from 2010 to 2020 were also reported. Since for some entities, the flow of exemptions for pathology is also available for the years prior to 2010, these patients were also considered. For each calendar year, the changes in incidence over time were investigated using Poisson regression, estimating the incidence rate ratios (IRRs) with 95% CIs.

The epidemiological data were reproportioned to the Italian population. For both prevalence and incidence, data were stratified by gender, by age classes at the index date (<18–50 years; >50 years) and by geographical distribution (northern, central and southern Italy).

Lastly, a 5-year projection of expected prevalence and incidence in the year 2027 was calculated.

### 2.4. Demographic and Clinical Characteristics of Adults with PNH

The analysis focused on the adult population with PNH. At the index date, patients’ demographic variables were recorded, namely, age reported in years and age groups (18–50 and >50 years) and gender presented as the proportion of male subjects. The comorbidity profile was investigated during the entire available period before the index date, searching for concomitant conditions identified using the ICD-9-CM for hospital discharge diagnoses (both primary and secondary diagnoses) and ATC codes for drug prescriptions. The comorbidity profile was evaluated by the Elixhauser Index, a scoring tool to estimate the overall weight of 30 comorbidities (reported with their ICD-9-CM codes in Table A1 of Appendix A) and predict the repercussions for hospital resource use and in-hospital mortality [23].

Before inclusion and during follow-up, adult patients with PNH were assessed for the presence of PNH-related comorbidities and clinical manifestations mostly described in the literature [6,24,25]. Lastly, data on past red blood cell (RBC) transfusions prior to the index date were collected, and patients were classified as transfusion-dependent or not-dependent.

The codes used to identify medications, diseases or procedures are provided in Table A2 of Appendix A.

### 2.5. Treatment Patterns

In the included population of adults with PNH, treatments with anti-complement therapy were identified. The comparisons were carried out between patients stratified by the presence/absence of anti-complement treatment with C5/3 inhibitors (C5/3i), identified by the ATC code L04AA (L04AA25: eculizumab; L04AA43: ravulizumab; L04AA54: pegcetacoplan).

### 2.6. Healthcare Resource Consumption and Direct Costs

Annualized HCRU and the related direct costs derived from the INHS were evaluated among living patients with at least 12 months of follow-up from the index date to the end of the available data. An in-depth analysis was conducted in the cohort of patients treated with anti-complement therapy to assess the yearly consumption and costs of C5/3i, considering two further time intervals: (i) from the date of the first prescription to the end of follow-up, and (ii) from the first prescription to the last prescription plus the treatment duration (actual treatment period).

HCRU was expressed as the annual number of services/resources delivered per patient in terms of drugs (anti-complement therapy, other PNH-related treatments and non-PNH-related treatments), specialist visits, diagnostic services and hospitalizations (all-cause, due to PNH-related comorbidities, due to PNH-related clinical manifestations, due to RBC transfusion, due to the infusion of anti-complement treatment). For hospitalizations, the length of stay was also computed. The healthcare direct costs derived from all the mentioned resource consumptions were also assessed and reported in euros (€). Specifically, hospitalizations were determined using the DRG tariffs [26], drug costs were evaluated using the INHS purchase price [27,28,29,30] and outpatient specialist service costs were estimated based on regional tariffs. Costs were calculated as overall expenditure (referring to the entire available follow-up and all the patients in each cohort) and per patient per year (PPPY).

An in-depth analysis was also conducted of the subset of patients treated with C5/3i to evaluate HCRU and healthcare direct costs considering two time intervals: from the start of treatment to the end of follow-up and from the start to the end of treatment.

### 2.7. Statistical Analysis

Continuous variables are expressed as mean and standard deviation (±SD) and categorical variables as frequencies and percentages. Continuous variables were compared using Student’s *t*-test and categorical variables by the chi-squared test or Fisher’s exact test (for expected frequencies < 5), as appropriate.

Results referring to subgroups of less than four patients were not disclosed and were reported as NI (not issuable) since they might be potentially traceable to single individuals, in line with the code for the protection of personal data (“Codice in materia di protezione dei dati personali”).

In cost analysis, the outliers (those values that deviate more than three times the SD) were excluded. A p value below 0.05 was considered statistically significant, and all the analyses were performed using STATA SE, version 17.0 (StataCorp LLC, College Station, TX, USA).

## 3. Results

### 3.1. Epidemiological Analysis of the Total Population (All Ages)

**Figure A1:**
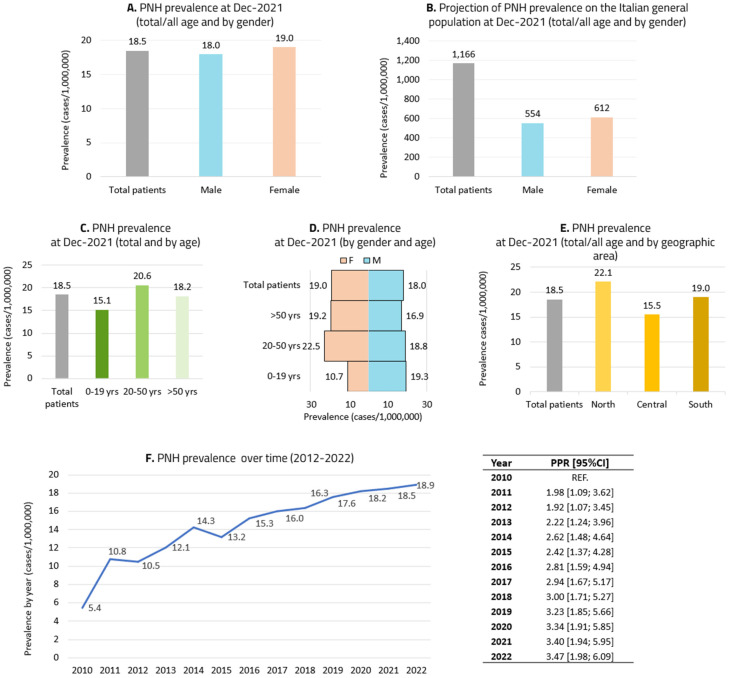
Prevalence of PNH in the total population (all ages): (**A**) total patients and stratified by gender; (**B**) data projected on the Italian population; (**C**) prevalence stratified by age; (**D**) by gender and age; (**E**) by geographic area of Italy; (**F**) by year (2010–2022).

**Figure A2:**
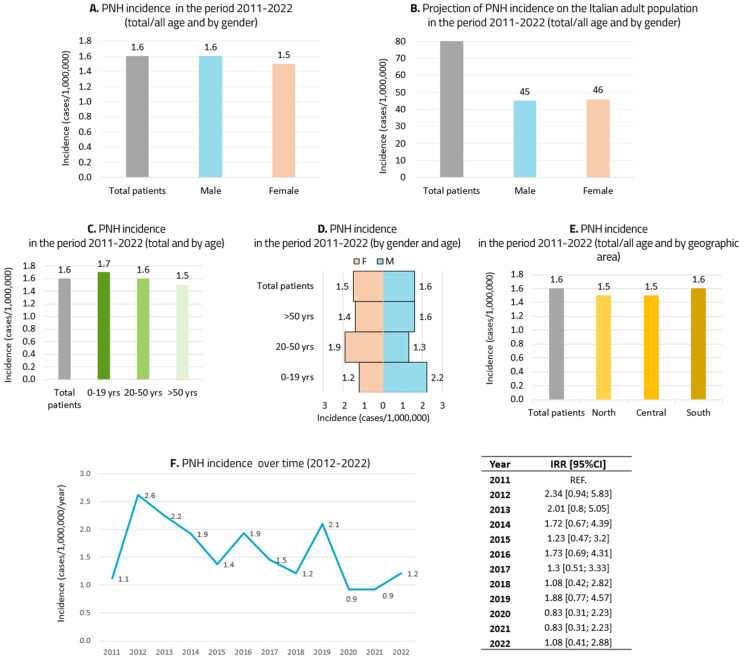
Incidence of PNH in the total population (all ages): (**A**) total patients and stratified by gender; (**B**) data projected on the Italian population; (**C**) prevalence stratified by age; (**D**) by gender and age; (**E**) by geographic area of Italy; (**F**) by year (2010–2022).

A first epidemiological analysis was conducted on the total PNH population of pediatric and adult subjects. The detailed results are provided in Figure A1 and Figure A2 of Appendix B.

The prevalence of PNH in 2021 was 18.5/1,000,000 people, with, specifically, 18.0/1,000,000 males and 19.0/1,000,000 females (Figure A1A of Appendix B), which, reproportioned to the Italian population, corresponded to 1,166 patients with prevalent PNH on 31 December 2021, of whom 554 were male and 612 female (Figure A1B of Appendix B). The analysis of prevalence stratified by age (Figure A1C of Appendix B) revealed that most of the patients with prevalent PNH were in the 20–50 age group (0–19 years: 51.1/1,000,000 people; 20–50 years: 20.6/1,000,000 people; and >50 years: 18.2 cases/1,000,000 people). The analysis of age and gender-stratified prevalence (Figure A1D of Appendix B) revealed that the proportion of males and females varied across age groups: in patients aged 0–19 years, the prevalence was 19.3 males/1,000,000 people vs. 10.7 females/1,000,000 people; in patients aged 20–50 years, the prevalence was 18.8 males/1,000,000 people vs. 22.5 females/1,000,000 people; in patients over 50 years, the prevalence was 16.9 males/1,000,000 people vs. 19.2 females/1,000,000 people. The prevalence stratified by geographic area (Figure A1E of Appendix B) was as follows: 22.1/1,000,000 people in northern Italy, 15.5/1,000,000 people in central Italy and 19.0/1,000,000 people in southern Italy. Prevalence in the timespan between 2010 and 2022 showed a rising trend (from 5.4/1,000,000 people in 2010 to 18.9/1,000,000 people in 2022) (Figure A1F of Appendix B). Projecting the data 5 years, the estimated prevalence rate of PNH is expected to be 19.9/1,000,000 people in 2027.

The incidence rate of PNH among the total population in 2021 was 1.6/1,000,000 people, with, specifically, 1.6/1,000,000 males and 1.5/1,000,000 females (Figure A2A of Appendix B), which, projected on a national scale, corresponded to 91 patients with incidence of PNH as of 31 December 2021, 45 male and 46 female (Figure A2B of Appendix B). The age-stratified incidence was 1.7/1,000,000 people for patients aged 0–19 years, 1.6 for those in the range of 20–50 years and 1.5 for those aged over 50 years (Figure A2C of Appendix B). The gender-stratified incidence confirmed variable proportions of males and females across age groups (Figure A2D of Appendix B): in patients aged 0–19 years, the incidence was 2.2 males/1,000,000 people vs. 1.2 females/1,000,000 people; in patients aged 20–50 years, the incidence was 1.3 males/1,000,000 people vs. 1.9 females/1,000,000 people; in patients over 50 years, the incidence was 1.6 males/1,000,000 people vs. 1.4 females/1,000,000 people. The incidence stratified by geographic area was 1.5/1,000,000 people in northern Italy, 1.5/1,000,000 people in central Italy and 1.6/1,000,000 people in southern Italy (Figure A2E of Appendix B). The incidence fluctuated over time, with a rising and descending course between 2011 and 2022 (Figure A2F of Appendix B). Projecting the data 5 years, the estimated incidence rate of PNH is expected to be 1.3/1,000,000 people in 2027.

### 3.2. Epidemiological Analysis in Adults

**Figure 1:**
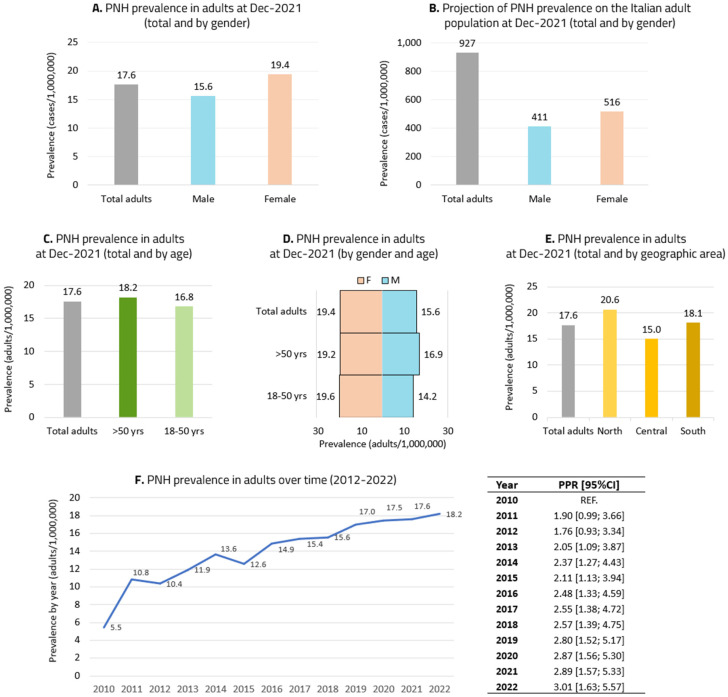
Prevalence of PNH among adults only: (**A**) total adults and stratified by gender; (**B**) data projected on the Italian population; (**C**) prevalence stratified by age; (**D**) prevalence stratified by gender and age; (**E**) prevalence stratified by geographic area of Italy; (**F**) prevalence stratified by year and prevalence proportion ratios with 95% CIs between 2010 and 2022.

The prevalence of PNH in the adult (>18 years) population in 2021 was 19.4/1,000,000 people, 15.6/1,000,000 males and 19.4/1,000,000 females (Figure 1A). Data reproportioned to the Italian population corresponded to 927 adult patients with prevalent PNH as of 31 December 2021, of whom 411 were male and 516 female (Figure 1B). Age-stratified prevalence revealed that patients with prevalent PNH constituted 16.8/1,000,000 people in the age range of 18–50 years and 18.2/1,000,000 people in the age group over 50 years (Figure 1C). Prevalence stratified by gender and age was 14.2 males/1,000,000 people vs. 19.2 females/1,000,000 people in patients aged 18–50 years and 16.9 males/1,000,000 people vs. 19.2 females/1,000,000 people in patients over 50 years (Figure 1D). Prevalence stratified by geographic area in adults with PNH was as follows: 20.6/1,000,000 people in northern Italy, 15.0/1,000,000 people in central Italy and 18.1/1,000,000 people in southern Italy (Figure 1E). Prevalence by year in the period between 2010 and 2022 showed a rising trend (from 5.5/1,000,000 people in 2010 to 18.2/1,000,000 people in 2022) (Figure 1F). Projecting the data 5 years, the expected prevalence of PNH is estimated to be 19.0/1,000,000 people in 2027.

**Figure 2:**
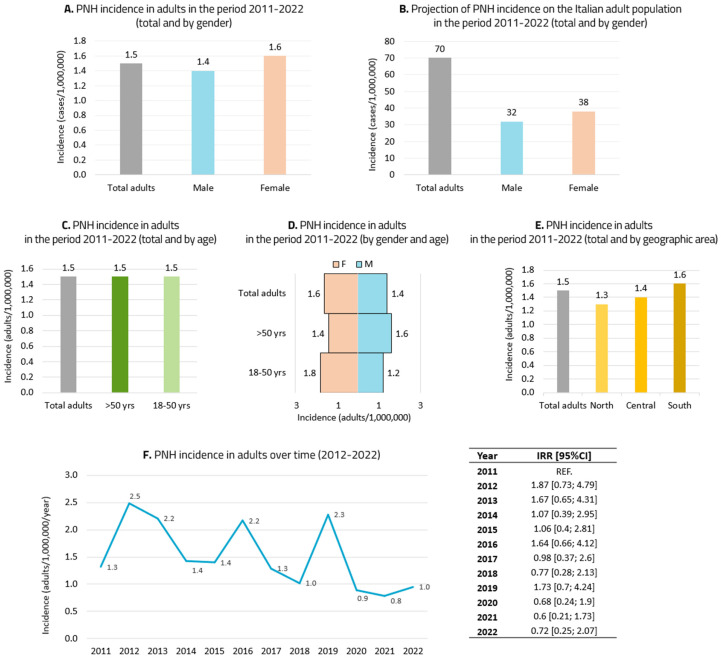
Incidence of PNH among adults only: (**A**) total adults and stratified by gender; (**B**) data projected on the Italian population; (**C**) incidence stratified by age; (**D**) incidence stratified by gender and age; (**E**) incidence stratified by geographic area of Italy; (**F**) incidence stratified by year (2011–2022).

The incidence of PNH in the adult population in 2021 was 1.5/1,000,000 people, 1.4/1,000,000 males and 1.6/1,000,000 females (Figure 2A). Data projected on the Italian population corresponded to 70 adults with an incidence of PNH on 31 December 2021, of whom 32 were male and 38 female (Figure 2B). The age-stratified incidence was 1.5/1,000,000 people in both age groups (Figure 2C). The incidence stratified by gender and age was as follows: 1.8 males/1,000,000 people vs. 1.2 females/1,000,000 people in the age range of 18–50 years and 1.6 males/1,000,000 people vs. 1.4 females/1,000,000 people over 50 years (Figure 2D). The incidence stratified by geographic area in adults with PNH was 1.3/1,000,000 people in northern Italy, 1.4/1,000,000 people in central Italy and 1.6/1,000,000 people in southern Italy (Figure 2E). The incidence in adult patients with PNH had a rising and descending course between 2011 and 2022 (Figure 2F). Projecting the data 5 years, the expected incidence of PNH is estimated to be 1.1/1,000,000 people in 2027.

### 3.3. Main Features of the Adult Population

**Figure 3:**
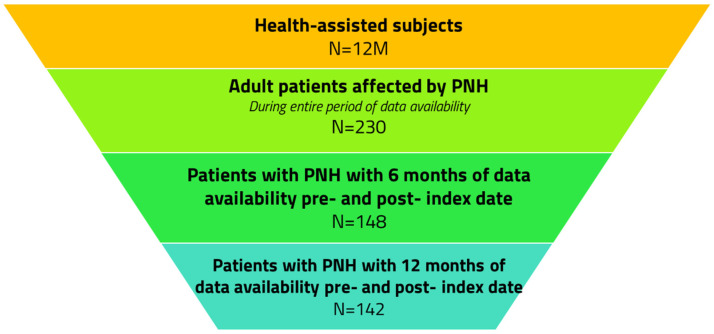
Flowchart of the included adult patients with PNH.

The demographics, clinical characteristics and therapy schedules of adults with PNH were then analyzed. As detailed in Figure 3, from a study sample covering approximately 20% of the Italian population, 230 adult patients diagnosed with PNH were identified, considering all available periods in the database. Of these 230 patients, 137 (60%) were entered into the analysis through the hospitalization code and 93 (40%) through the exemption code. Among them, 148 had at least 6 months of data available before and after the index date, and 142 had at least 12 months of data available before and after the index date.

Among the 142 patients identified with PNH with 12 months of available data before and after the index date, 38 (27%) received anti-complement therapy with C5/3i, which was more commonly prescribed to younger patients (treated patients aged 18–50 years: 63.2%; treated patients aged > 50 years: 36.8%). The mean (±SD) follow-up was 5.9 (±3.2) years in the total PNH population, 7.0 (±3.3) years in patients with PNH treated with C5/3i therapy and 5.5 (±3.1) years in the untreated population. The time from the index date to the start of anti-complement therapy was 2.8 years, all patients started with eculizumab treatment and 13.2% of the treated patients switched to another C5/3i drug (five patients switched to either ravulizumab (n ≤ 4) or pegcetacoplan (n ≤ 4)). The median duration of treatment (considering all treatments before and after the switch) was 3.4 years (range: 0.0–12.0 years).

As detailed in Table 1, the 142 adults with PNH included in the analysis had an average age at the index date of 50.7 years, and there was a slight predominance of females (males: 45.8%). When comparing patients stratified by treatment with anti-complement therapy, younger patients (18–50 years) were more represented among the treated (63.2%) and older patients among the untreated (55.8%), although the difference was nearly significant (*p* = 0.077).

The majority of included patients came from the healthcare entities of southern Italy, followed by central and northern Italy, with 65.5%, 23.9% and 10.6%, respectively (Table 1).

The mean (±SD) Elixhauser Comorbidity Index in all adult patients with PNH during the 12 months before the index date was 1.4 (±4.1).

Then, PNH-related comorbidities and the most frequent clinical manifestation of PNH were analyzed during the pre-index characterization period, with a mean (±SD) length of 5.0 (±3.0) years in all adults with PNH, 3.7 (±2.4) years in those treated with C5/3i and 5.5 (±3.0) years in those not treated with C5/3i (median: 4.5, 2.8 and 5.0 years, respectively).

As Table 2A shows, the most common comorbidities detected during the characterization period were AA and other BMF syndromes (found in 10.6% of all patients with PNH) and MDS (found in 4.2% of all patients with PNH). Comparing patients treated and not treated with C5/3i, there were no significant differences in the distribution of comorbidities, although AA and other BMF syndromes were moderately more represented in the group of treated patients (18.4% vs. 7.7%, *p* = 0.174).

As Table 2B shows, the clinical manifestation of PNH did not differ significantly across groups, although anemia (excluding AA) was moderately more frequent (not significant) among the patients treated with C5/3i vs. those untreated (28.9% vs. 13.5%, *p* = 0.101).

Of the patients treated with anti-complement therapy, 35.5% had undergone at least one RBC transfusion in the 6 months before starting C5/3i therapy. Among them, 33.3% had successive transfusions after 6, 12 and 18 months of anti-complement therapy initiation and 30.8% after 24 months.

Then, PNH-related comorbidities and the most frequent clinical manifestation of PNH were analyzed during the entire available follow-up, with a mean (±SD) length in years of 5.9 (±3.2) in all adults with PNH, 7.0 (±3.3) in those treated with C5/3i and 5.5 (±3.1) in those not treated with C5/3i (median: 5.2, 6.6 and 5.1 years, respectively). 

As Table 3A shows, the most common comorbidities detected during follow-up were AA and other BMF syndromes (found in 15.5% of all patients with PNH). The comparisons of patients treated vs. untreated with C5/3i revealed a significantly higher frequency in the group of treated patients vs. the untreated of AA and other BMF syndromes (28.9% vs. 10.6%, *p* = 0.043) and of MDS (13.2% vs. NI (≤4 patients), *p* = 0.028). Concerning the clinical manifestation of PNH detailed in Table 3B, anemia (excluding AA) was the most common (found in 38.7%, 47.4% and 35.6% of all patients with PNH, patients treated with C5/3i and those untreated, respectively). The patterns of clinical manifestation of PNH were comparable between patients treated and not treated with C5/3i.

A detailed description of the 10 most frequent treatments prescribed (other than anti-complement therapy) and 10 most frequent causes of hospitalization during follow-up are listed in Table 4A and 4B, respectively. The largest proportion of patients with PNH used amoxicillin with an enzyme inhibitor (58.8%), followed by folic acid (41.9%), pantoprazole (41.9%) and prednisone (41.2%). Eculizumab was the most prescribed agent in patients under anti-complement therapy. Blood and blood-forming organs and immunological disorders were the most frequent causes of hospitalization in the whole population, regardless of treatment or not with C5/3i, followed by circular system and kidney/urinary tract complications.

### 3.4. Healthcare Resource Consumption in the Adult Population

HCRU was analyzed per patient per year among patients with at least 12 months of follow-up, with a mean follow-up of 6.1 years (median: 5.5 years) for all patients with PNH, 7.0 years (median: 6.6 years) for the patients treated with C5/3i and 5.8 years (median: 5.2 years) for untreated patients (Table 5). The comparison of patients treated and untreated with C5/3 inhibitors showed that those who received anti-complement therapy had a significantly higher number of specialist visits, diagnostic tests and hospitalizations for transfusions as well as longer hospital stays.

#### Focused Analysis of Patients Treated with C5/3i: Healthcare Resource Consumption

HCRU was then analyzed per patient per year among 38 patients treated with C5/3i, based on the interval from the start to the end of treatment, which was on average 4.1 years (median: 3.4 years—Table 6).

### 3.5. Healthcare Direct Costs in the Adult Population

The direct annual costs (excluding outliers) during follow-up, overall and per patient, in all adults with PNH and after stratification by the presence/absence of treatment with C5/3i are detailed in Table 7. The overall average cost per patient per year (PPPY) for the management of adults with PNH was EUR 41,084 (median: EUR 3976), distributed as EUR 35,839 (median: EUR 837) for drugs, EUR 4284 (median: EUR 986) for hospitalizations and EUR 962 (median: EUR 209) for outpatient specialist services. The mean drug expenses (per patient per year) consisted of EUR 32,816 (median: EUR 0) for anti-complement therapy, EUR 631 (median: EUR 13) for other PNH-related treatments and EUR 2391.5 (median: EUR 209) for other therapies. The costs for hospitalizations were mainly driven by RBC transfusions (mean: EUR 1983; median: EUR 0), followed by other hospitalizations (mean: EUR 1087; median: EUR 54), PNH comorbidities (mean: EUR 662; median: EUR 0) and PNH clinical manifestations (mean: EUR 552; median: EUR 0).

The healthcare costs per patient per year during follow-up were then compared for PNH stratified by the presence/absence of treatment with C5/3i. The treated cohort was associated with higher total costs (EUR 133,472 vs. EUR 8089, *p* < 0.001), mainly due to drug expenses (EUR 127,180 vs. EUR 3217, *p* < 0.001). In the group of patients treated with C5/3i, considering the interval from anti-complement therapy initiation to the last prescription plus the treatment period, the annual direct costs per patient for C5/3i prescriptions were EUR 290,256.7 (± EUR 56,165.6) and EUR 290,256.7 (EUR 56,165.6), expressed, respectively, as the mean (±SD) and median (IQR).

#### Focused Analysis of Patients Treated with C5/3i: Healthcare Costs

Costs were analyzed per patient per year for 37 patients (one outlier excluded) treated with C5/3i during the period from the start to the end of treatment (Table 8).

## 4. Discussion

PNH is a rare disease, and epidemiological data in Italy and worldwide are vague. Thus, one main purpose of this real-world analysis was to provide an updated picture of the numbers of patients with PNH in Italy with a special focus on adult patients’ distribution according to age, gender and geographic area. Although PNH can develop at any age, our data identified a relatively young population, in line with the literature [3,14] and international registries [6,31]. Given the lack of data in Italy, especially stratified by latitude, our data did not highlight noticeable differences in the epidemiology of PNH between northern, central and southern Italy and were largely consistent with estimates from other European and extra-European countries [3,13]. In this analysis, we found that the yearly PNH prevalence showed moderately rising numbers, with an overall fluctuating course of incidence rates. Similarly, data on patients with acquired hemolytic disorders collected between 1977 and 2016 from the Danish National Patient Register reported increased incidence and prevalence rates over time for all types of related disorders except drug-induced hemolysis [14]. For PNH, the estimated prevalence per 100,000 people grew from 0.18 in 1980 to 1.04 in 2015 [14]. A UK study focused on PNH with detectable clones showed a prevalence and yearly incidence in the UK between 2004 and 2018 of 38 and 3.5 per million people, respectively [3]. Possible differences between the currently available reports from various countries and with respect to our epidemiological estimates might be related to the methods used to identify the diagnosis and PNH classification [10,11,12,13].

The present analysis then focused on the characterization of adult patients with PNH and their therapeutic journey. However, it should be specified that the use of administrative databases does not allow discrimination between the different forms of PNH since they are all coded with the same ICD-9-CM code. PNH is a highly heterogeneous disease; thus, the different treatment algorithm recommended for each clinical form might be the reason for the relatively small proportion of patients prescribed with complement inhibitor therapy [6]. Based on the positive data from clinical trials, eculizumab was the first authorized C5 inhibitor and represents a major advance in the therapeutic landscape of PNH [27,28]. A phase-III clinical trial published on 87 patients with PNH reported better outcomes in the eculizumab-treated group than in the placebo group in terms of hemoglobin stabilization without needing transfusion and quality of life [27]. These data were confirmed by a 52-week, phase-III, open-label study evaluating eculizumab in 97 patients with PNH across 33 international sites. Eculizumab significantly reduced hemolysis, improved fatigue scores and increased hemoglobin levels while reducing the number of transfusions [28].

Successively, ravulizumab was approved for PNH on the basis of two phase-III trials. The ALXN1210-PNH-301 study involved 246 patients who were treatment-naïve and showed that ravulizumab was as effective as eculizumab in controlling hemolysis and reducing the number of transfusions [29]. The ALXN1210-PNH-302 study included 195 patients who were stable on eculizumab and demonstrated that ravulizumab maintained disease control with a less frequent dosing schedule. In both trials, ravulizumab provided a longer-acting treatment option, reducing the treatment burden for patients while ensuring successful disease management [30].

Pegcetacoplan is the most recent complement inhibitor, introduced to clinical practice for PNH treatment based on the results of the PEGASUS trial. This phase-III study assessed pegcetacoplan in 80 patients with PNH with persistent anemia despite eculizumab treatment. The results showed that pegcetacoplan was associated with improved hemoglobin, clinical and hematologic outcomes and was generally a broader hemolysis control than eculizumab [32].

In Italy, eculizumab, ravulizumab and pegcetacoplan are currently approved and used in clinical practice for the treatment of PNH [33,34,35,36]. While eculizumab and ravulizumab are also indicated for atypical hemolytic uremic syndrome (aHUS), pegcetacoplan is approved for patients with PNH who remain anemic despite C5 inhibitor therapy. The prescription and management of these therapies are typically restricted to specialized centers with expertise in rare hematologic disorders.

Despite the subsequent approval of ravulizumab [34] and pegcetacoplan [35], there are still unmet needs related to efficacy, safety and manageability that have not been solved, including persistent anemia, transfusion dependence, fatigue and health-related quality of life (HRQoL) impairment [31,37,38].

C5 inhibitors do not allow complete PNH control, which can only be achieved by preventing intravascular and extravascular hemolysis to ensure the normalization of hemoglobin, a reduction in LDH, transfusion independence and a reduction in fatigue to levels observed in the general population [39]. Our findings seem to corroborate this view—among the 38 adults with PNH who received anti-complement therapy with C5/3i, about one-third required successive transfusions within 18 months after anti-complement therapy initiation and 30.8% after 24 months, suggesting the need for other treatment options that act on the early phases of complement activation and are able to control C3-mediated extravascular hemolysis [37].

The results of the analysis of healthcare consumption and the related costs should take into consideration the clinically heterogenous forms of PNH and the impossibility of discriminating between them using administrative databases. Despite this flaw, it is feasible that, in line with the recommendations, complement inhibitors were prescribed to patients with the classical disease form, and their more complex clinical status might explain the larger requirement for hospitalizations, transfusion and outpatient services [24,39]. Recently, it has been reported that a patient-tailored dosing strategy might be useful to improve the cost-effectiveness of expensive anti-complement drugs, also allowing cost savings compared to eculizumab [40,41].

The present results must be interpreted considering some limitations related to the observational design of the analysis, which utilized data extracted from administrative databases. First, the use of ICD-9-CM codes represents a diagnosis proxy to extrapolate patients with PNH and does not allow differentiation between the different disease forms, which have a different clinical course and, consequently, different therapeutic management. Besides the advantages due to the large, unselected sample size of subjects, representing around one-fifth of the Italian population, administrative flows might lack some information that cannot be traced within these repositories meant for reimbursement purposes. Therefore, not all the services or treatments covered by the INHS were able to be retrieved, and their weight as potential confounders was not evaluated. Moreover, reasons beyond blood transfusion requirements not directly related to PHN itself or diagnosis delays could not be traced. The comorbidities included in the calculation of the CEI were identified in the databases by means of hospital discharge codes or drug prescriptions as a proxy of diagnosis; so, there was no way to assess the severity and the actual burden of each concomitant condition. Moreover, the databases of the various participating entities did not have uniform completeness, so the time intervals of the epidemiological estimates were not the same—in some cases, data were available back to 2011 and in other cases back to 2010 or earlier. The majority of included patients came from the healthcare entities of southern Italy, followed by central and northern Italy (65.5%, 23.9% and 10.6%, respectively). Such a disparity might be explained by the fact that only some healthcare institutions included in the analysis are reference centers for this rare condition. Although no substantial difference in PNH epidemiology emerged between the geographical areas, the current findings should be confirmed on a larger population with a balanced distribution across the national territory. Lastly, the lack of a clinical hematologist in the research group might have constrained the interpretation of specific findings, particularly regarding therapeutic implications.

In conclusion, the present real-world data analysis conducted in Italy estimated the number of patients with a diagnosis of PNH, confirming moderately rising numbers in terms of prevalence and good consistency with European and global estimates. The benefits of anti-complement therapy, especially regarding agents targeted at C3 and C5, seem to not be fully exploited yet, and several patients still retain a substantial burden of illness. Thus, the hematological response in patients with PNH might benefit from novel therapeutics targeted at upstream complement inhibition.

## Figures and Tables

**Table 1:** Demographic and clinical characteristics of adult patients with PNH: total and stratified by the presence/absence of treatment with C5/3i. The *p* values refer to comparisons between patients treated with C5/3i (n = 38) and patients not treated with C5/3i (n = 104).

Demographic and Clinical Variables	Total Adults (n = 142)	Patients Treated with C5/3i (n = 38)	Patients Not Treated with C5/3i (n = 104)	*p*
Age at index date, years, mean (±SD)	50.7 (±17.9)	47.6 (±14.1)	51.9 (±18.6)	0.259
Age at index date, years, median (range)	51 (19–94)	43 (28–79)	52 (19–94)	
Age stratification				
18–50 years, n (%)	70 (49.3%)	24 (63.2%)	46 (44.2%)	0.077
>50 years, n (%)	72 (50.7%)	14 (36.8%)	58 (55.8%)	
Males, n (%)	65 (45.8%)	16 (42.1%)	49 (47.1%)	0.729
Geographical distribution				
North, n (%)	15 (10.6%)	5 (13.2%)	10 (9.6%)	0.153
Central, n (%)	34 (23.9%)	14 (36.8%)	20 (19.2%)	
South, n (%)	93 (65.5%)	19 (50.0%)	74 (71.2%)	

Abbreviations: SD, standard deviation.

**Table 2:** PNH-related comorbidities (**A**) and main clinical manifestations (**B**) during the characterization period in adult patients with PNH: total and stratified by the presence/absence of treatment with C5/3i. The *p* values refer to comparisons between patients treated with C5/3i (n = 38) and not treated with C5/3i (n = 104). Significances are highlighted in bold.

A. PNH-Related Comorbidities During the Characterization Period, n (%)	Total Adults (n = 142)	Patients Treated with C5/3i (n = 38)	Patients Not Treated with C5/3i (n = 104)	*p*
AA and other BMF syndromes	15 (10.6%)	7 (18.4%)	8 (7.7%)	0.176
MDS	6 (4.2%)	4 (10.5%)	NI	0.091
Hematological malignancy	NI	NI	NI	0.485
Acute myelogenous leukemia	0 (0%)	0 (0%)	0 (0%)	/
Myeloproliferative disorder	0 (0%)	0 (0%)	0 (0%)	/
Myelofibrosis	0 (0%)	0 (0%)	0 (0%)	/
None	118 (83.1%)	26 (68.4%)	92 (88.5%)	**0.016**
**B. Clinical Manifestations of PNH During the Characterization Period, n** (**%**)				
Anemia (excluding AA)	25 (17.6%)	11 (28.9%)	14 (13.5%)	0.101
Thrombosis	5 (3.5%)	0 (0%)	5 (4.8%)	0.583
Chronic kidney disease	5 (3.5%)	0 (0%)	5 (4.8%)	0.583
Major adverse vascular events	NI	0 (0%)	NI	1.000
Abdominal pain	0 (0%)	0 (0%)	0 (0%)	/
Dysphagia	0 (0%)	0 (0%)	0 (0%)	/
Dyspnea	0 (0%)	0 (0%)	0 (0%)	/
Erectile dysfunction	0 (0%)	0 (0%)	0 (0%)	/
Fatigue	0 (0%)	0 (0%)	0 (0%)	/
Pulmonary hypertension	0 (0%)	0 (0%)	0 (0%)	/
None	105 (73.9%)	27 (71.1%)	78 (75.0%)	0.662

Abbreviations: AA, aplastic anemia; BMF, bone marrow failure; MDS, myelodysplastic syndrome; NI, not issuable; PNH, paroxysmal nocturnal hemoglobinuria.

**Table 3:** PNH-related comorbidities (**A**) and main clinical manifestations (**B**) during the entire follow-up period in adult patients with PNH: total and stratified by the presence/absence of treatment with C5/3i. The *p* values refer to comparisons between patients treated with C5/3i (n = 38) and patients not treated with C5/3i (n = 104). Significances are highlighted in bold.

A. PNH-Related Comorbidities During Characterization Period, n (%)	Total Adults (n = 142)	Patients Treated with C5/3i (n = 38)	Patients Not Treated with C5/3i (n = 104)	*p*
AA and other BMF syndromes	22 (15.5%)	11 (28.9%)	11 (10.6%)	**0.043**
MDS	7 (4.9%)	5 (13.2%)	NI	**0.028**
Hematological malignancy	5 (3.5%)	NI	4 (3.8%)	1.000
Acute myelogenous leukemia	NI	0 (0%)	NI	1.000
Myeloproliferative disorder	0 (0%)	0 (0%)	0 (0%)	/
Myelofibrosis	0 (0%)	0 (0%)	0 (0%)	/
None	107 (75.4%)	21 (55.3%)	86 (82.7%)	**0.001**
**B. Clinical Manifestations of PNH During Characterization Period, n** (**%**)				
Anemia (excluding AA)	55 (38.7%)	18 (47.4%)	37 (35.6%)	0.351
Thrombosis	6 (4.2%)	4 (10.5%)	NI	0.091
Chronic kidney disease	11 (7.7%)	NI	10 (9.6%)	0.693
Major adverse vascular events	6 (4.2%)	NI	NI	0.338
Abdominal pain	NI	NI	NI	0.485
Dysphagia	0 (0%)	0 (0%)	0 (0%)	/
Dyspnea	0 (0%)	0 (0%)	0 (0%)	/
Erectile dysfunction	0 (0%)	0 (0%)	0 (0%)	/
Fatigue	0 (0%)	0 (0%)	0 (0%)	/
Pulmonary hypertension	NI	NI	NI	0.357
None	59 (41.5%)	10 (26.3%)	49 (47.1%)	**0.032**

Abbreviations: AA, aplastic anemia; BMF, bone marrow failure; MDS, myelodysplastic syndrome; NI, not issuable; PNH, paroxysmal nocturnal hemoglobinuria.

**Table 4:** (**A**) Ten most frequent prescriptions (excluding C5/3i) and (**B**) 10 most frequent causes of hospitalization during follow-up in patients with PNH: total and stratified by the presence/absence of treatment with C5/3i.

A. 10 Most Frequent Drugs (Excluding C5/3i)					
Total Adults (n = 136)		Patients Treated with C5/3i (n = 38)		Patients Not Treated with C5/3i (n = 98)	
Amoxicillin and enzyme inhibitor	80 (58.8%)	Amoxicillin and enzyme inhibitor	26 (68.4%)	Amoxicillin and enzyme inhibitor	54 (55.1%)
Folic acid	57 (41.9%)	Folic acid	26 (68.4%)	Pantoprazole	43 (43.9%)
Pantoprazole	57 (41.9%)	Ciprofloxacin	23 (60.5%)	Prednisone	41 (41.8%)
Prednisone	56 (41.2%)	Levofloxacin	20 (52.6%)	Cholecalciferol	35 (35.7%)
Ciprofloxacin	53 (39.0%)	Enoxaparin	16 (42.1%)	Furosemide	33 (33.7%)
Levofloxacin	50 (36.8%)	Prednisone	15 (39.5%)	Enoxaparin	31 (31.6%)
Enoxaparin	47 (34.6%)	Beclomethasone	15 (39.5%)	Folic Acid	31 (31.6%)
Cholecalciferol	45 (33.1%)	Cefixime	14 (36.8%)	Azithromycin	31 (31.6%)
Azithromycin	42 (30.9%)	Rifaximin	14 (36.8%)	Ciprofloxacin	30 (30.6%)
Furosemide	41 (30.1%)	Pantoprazole	14 (36.8%)	Levofloxacin	30 (30.6%)
**B. 10 Most Frequent Hospitalizations**					
**Total Adults** (**n = 136**)		**Patients Treated with C5/3i** (**n = 38**)		**Patients Untreated with C5/3i** (**n = 98**)	
Blood and blood-forming organs and immunological disorders	76 (55.9%)	Blood and blood-forming organs and immunological disorders	26 (68.4%)	Blood and blood-forming organs and immunological disorders	50 (51.0%)
Circulatory system	21 (15.4%)	Kidney and urinary tract	12 (31.6%)	Circulatory system	16 (16.3%)
Kidney and urinary tract	18 (13.2%)	Infectious and parasitic DDs	8 (21.1%)	Respiratory system	11 (11.2%)
Hepatobiliary system and pancreas	15 (11%)	Myeloproliferative DDs (poorly differentiated neoplasms)	5 (13.2%)	Hepatobiliary system and pancreas	10 (10.2%)
Infectious and parasitic DDs	14 (10.3%)	Hepatobiliary system and pancreas	5 (13.2%)	Musculoskeletal system and connective tissue	7 (7.1%)
Respiratory system	13 (9.6%)	Circulatory system	5 (13.2%)	Digestive system	7 (7.1%)
Digestive system	10 (7.4%)	Mental diseases and disorders	4 (10.5%)	Infectious and parasitic DDs	6 (6.1%)
Factors influencing health status	9 (6.6%)	Female reproductive system	4 (10.5%)	Kidney and urinary tract	6 (6.1%)
Pregnancy	9 (6.6%)	Digestive system	NI	Pregnancy	6 (6.1%)
Myeloproliferative DDs (poorly differentiated neoplasms)	9 (6.6%)	Factors influencing health status	NI	Factors influencing health status	6 (6.1%)
Blood and blood-forming organs and immunological disorders	76 (55.9%)	Blood and blood-forming organs and immunological disorders	26 (68.4%)	Blood and blood-forming organs and immunological disorders	50 (51.0%)
Circulatory system	21 (15.4%)	Kidney and urinary tract	12 (31.6%)	Circulatory system	16 (16.3%)
Kidney and urinary tract	18 (13.2%)	Infectious and parasitic DDs	8 (21.1%)	Respiratory system	11 (11.2%)
Hepatobiliary system and pancreas	15 (11%)	Myeloproliferative DDs (poorly differentiated neoplasms)	5 (13.2%)	Hepatobiliary system and pancreas	10 (10.2%)

Abbreviations: DDs, diseases and disorders. NI, not issued.

**Table 5:** Annual HCRU per patient with the average length of hospital stays during follow-up in adult patients with PNH: total and stratified by the presence/absence of treatment with C5/3i. The *p* values refer to comparisons between patients treated with C5/3i (n = 38) and patients not treated with C5/3i (n = 98). Significances are highlighted in bold.

	Total PNH Adults	Patients Treated with C5/3i	Patients Not Treated with C5/3i	*p*
Post-index period, years, mean (±SD)	6.1 (±3.1)	7.0 (±3.3)	5.8 (±3.0)	
median (IQR)	5.5 (1.0–12.8)	6.6 (1.0–12.1)	5.2 (1.0–12.8)	
**N. of annual HCRU per patient, mean** (**±SD**) *				
N. of PNH-related treatments	2.7 (±5.6)	9.5 (±9.1)	-	/
N. of other PNH-related treatments	3.7 (±5.1)	4.5 (±5.1)	3.4 (±5.2)	0.314
N. of non-PNH-related treatments	12.6 (±12.6)	11.7 (±11.6)	13.0 (±12.9)	0.632
N. of visits	2.9 (±4.9)	5.9 (±8.4)	1.7 (±2.8)	**<0.001**
N. of diagnostic services	3.4 (±4.9)	6.5 (±7.7)	2.2 (±3.4)	**<0.001**
N. of all-cause hospitalizations	1.0 (±1.7)	1.4 (±2.6)	0.8 (±1.3)	0.077
N. of hospital. for PNH-related comorbidities	0.3 (±1.2)	0.6 (±2.2)	0.2 (±0.7)	0.097
N. of hospital. for PNH-related clinical manifestations	0.3 (±0.6)	0.4 (±0.8)	0.3 (±0.5)	0.422
N. of hospitalizations for RBC transfusion	0.4 (±1.3)	0.8 (±2.5)	0.2 (±0.5)	**0.046**
**Length of hospitalization, days, mean** (**SD**)				
Length of all-cause hospitalization	60.0 (±89.8)	104.3 (±128.6)	46.0 (±68.8)	**0.004**
Length of hospital. for PNH-related comorbidities	91.4 (±107.8)	145.1 (±137.2)	65.7 (±82.1)	**0.043**
Length of hospital. for PNH-related clinical manifestations	49.5 (±87.0)	112.3 (±145.9)	30.6 (±47.1)	**0.001**

* The amount of annual HCRU per patient was calculated based on the post-index period. Abbreviations: HCRU, healthcare resource use; IQR, interquartile range; RBC, red blood cell; PNH, paroxysmal nocturnal hemoglobinuria; SD, standard deviation.

**Table 6:** Annual HCRU per patient per year among 38 patients treated with C5/3i, based on the period from the start to the end of treatment.

	From the Start to the End of Treatment (n = 38)
Post-index period, years, mean (±SD); median (IQR)	4.1 (±3.3); 3.4 (0.0–12.0)
**N. of annual HCRU per patient, mean** (**SD**)	
N. of PNH-related treatments	19.2 (±7.9)
N. of other PNH-related treatments	4.8 (±6.6)
N. of non-PNH-related treatments	10.9 (±12.0)
N. of visits	4.7 (±6.7)
N. of diagnostic services	5.9 (±7.9)
N. of all-cause hospitalizations	2.3 (±9.4)
N. of hospital. for PNH-related comorbidities	1.9 (±9.4)
N. of hospital. for PNH-related clinical manifestations	1.1 (±4.7)
N. of hospitalizations for RBC transfusion	2.0 (±9.4)

Abbreviations: HCRU, healthcare resource use; IQR, interquartile range; PNH, paroxysmal nocturnal hemoglobinuria; RBC, red blood cell; SD, standard deviation.

**Table 7:** Annual healthcare costs (overall and per patient) during follow-up in adults with PNH: overall and stratified by the presence/absence of treatment with C5/3i. Outliers were excluded from the analysis. The *p* values refer to the comparisons between patients treated with C5/3i (n = 38) and patients not treated with C5/3i (n = 98). Significances are highlighted in bold.

	Total PNH Adults (n = 133)		Patients Treated with C5/3i (n = 35)		Patients Not Treated with C5/3i (n = 98)		*p*
**A. Healtdcare costs** (**EUR**)	**Overall**	**PPPY**	**Overall**	**PPPY**	**Overall**	**PPPY**	
Total costs, mean (±SD)	280,378.7 (±493,973.4)	41,084.3 (±63,859.6)	964,728.2 (±772,651.5)	133,471.8 (±89,191.1)	35,968.1 (±58,819.1)	8088.7 (±16,244.0)	**<0.001**
Total costs, median (IQR)	21,615.1 (93,745.1)	3976.3 (21,495.1)	1,071,328.9 (907,322.7)	124,596.7 (133,564.4)	16,477.4 (30,170.6)	2324.5 (7029.8)	
Hospitalizations, mean (±SD)	16,592.8 (±28,589.0)	4283.6 (±11,699.8)	22,350.8 (±34,957.8)	5137.7 (±10,104.3)	14,536.4 (±26,881.3)	3978.5 (±12,071.2)	0.657
Hospitalizations, median (IQR)	4113.5 (17,077.8)	986.4 (3328.8)	5230.0 (38,741.2)	710.5 (3814.1)	3738.0 (14,942.7)	986.4 (2575.0)	
Drug prescriptions, mean (±SD)	257,851.9 (±490,905.3)	35,838.8 (±63,354.8)	934,672.4 (±776,562.4)	127,180.2 (±93,438.2)	16,130.3 (±44,205.4)	3216.9 (±8822.9)	**<0.001**
Drug prescriptions, median (IQR)	4635.1 (27,659.2)	836.7 (5989.5)	1,054,551.8 (917,433.9)	121,791.4 (146,686.4)	2163.3 (11,858.1)	442.9 (1775.1)	
Specialist services, mean (±SD)	5933.9 (±18,284.1)	961.9 (±2212.7)	7705.0 (±10,586.8)	1153.9 (±1452.3)	5301.4 (±19,651.5)	893.3 (±2356.7)	0.598
Specialist services, median (IQR)	1247.7 (3340.5)	209.3 (656.6)	2912.3 (8382.9)	556.5 (1147.7)	973.2 (2915.7)	170.7 (536.6)	
**B. Drug costs** (**EUR**)	**Overall**	**PPPY**	**Overall**	**PPPY**	**Overall**	**PPPY**	
Total drug costs, mean (±SD)	257,851.9 (±490,905.3)	35,838.8 (±63,354.8)	934,672.4 (±776,562.4)	127,180.2 (±93,438.2)	16,130.3 (±44,205.4)	3216.9 (±8822.9)	**<0.001**
Total drug costs, median (IQR)	4635.1 (27,659.2)	836.7 (5989.5)	1,054,551.8 (917,433.9)	121,791.4 (146,686.4)	2163.3 (11,858.1)	442.9 (1775.1)	
C5/3i, mean (±SD)	242,198.7 (±61,303.3)	32,815.9 (±10,874.3)	920,355.0 (±780,510.4)	124,700.6 (±94,641.6)	0.0 (±0.0)	0.0 (±0.0)	-
C5/3i, median (IQR)	0.00 (0.0)	0.0 (0.0)	1,053,352.2 (914,163.1)	112,468.0 (159,766.7)	0.0 (0.0)	0.0 (0.0)	
Other PNH-related drugs, mean (±SD)	3541 (±1460.4)	631.3 (±367.0)	4313.2 (±17,796.4)	482.1 (±1745.6)	3265.2 (±11,466.8)	684.6 (±2307.8)	0.681
Other PNH-related drugs, median (IQR)	74.05 (419.7)	13.4 (88.3)	92.20 (407.4)	42.4 (100.7)	70.48 (289.9)	10.7 (67.5)	
Other drugs, mean (±SD)	12,112.2 (±454,022.2)	2391.5 (±56,384.2)	10,004.2 (±22,862.7)	1997.6 (±4817.4)	12,865.0 (±38,195.5)	2532.2 (±7554.9)	0.736
Other drugs, median (IQR)	1330.12 (5885.7)	309.1 (1267.0)	1000.8 (5865.0)	119.9 (770.0)	1527.93 (5824.2)	372.9 (1405.6)	
**C. Hospitalization costs** (**EUR**)	**Overall**	**PPPY**	**Overall**	**PPPY**	**Overall**	**PPPY**	
Tot. hospitalization costs, mean (±SD)	16,592.8 (±28,589.0)	4283.6 (±11,699.8)	22,350.8 (±34,957.8)	5137.7 (±10,104.3)	14,536.4 (±26,881.3)	3978.5 (±12,071.2)	0.657
Tot. hospitalization costs, median (IQR)	4,113.5 (17,077.8)	986.4 (3328.8)	5230.0 (38,741.2)	710.5 (3814.1)	3738.0 (14,942.7)	986.4 (2575.0)	
PNH comorbidities, mean (±SD)	1914 (±9276.2)	662.2 (±3819.8)	1095.4 (±2873.5)	471.9 (±1883.6)	2206.4 (±10,821.8)	730.1 (±4302.9)	0.770
PNH comorbidities, median (IQR)	0.00 (0.0)	0.0 (0.0)	0.00 (0.0)	0.0 (0.0)	0.00 (0.0)	0.0 (0.0)	
PNH clinical manifest., mean (±SD)	2772.4 (±2157.6)	551.9 (±612.7)	3491.9 (±8255.7)	400.8 (±876.5)	2515.5 (±4744.1)	605.8 (±1538.0)	0.522
PNH clinical manifest., median (IQR)	0.00 (3738.0)	0.0 (498.4)	0.00 (1942.0)	0.0 (384.5)	0.00 (3738.0)	0.0 (507.9)	
RBC transfusions, mean (±SD)	5891 (±11,761.0)	1982.8 (±7201.9)	7290.4 (±15,880.6)	2665.8 (±9560.5)	5391.2 (±17,814.6)	1738.9 (±9645.3)	0.665
RBC transfusions, median (IQR)	0.00 (4264.0)	0.0 (851.9)	0.00 (4264.0)	0.0 (1310.7)	0.00 (4009.0)	0.0 (828.8)	
Other hospitalizations, mean (±SD)	6015.4 (±20,891.5)	1086.7 (±4689.3)	10,473.1 (±18,721.9)	1599.2 (±2453.1)	4423.3 (±10,258.6)	903.7 (±2385.0)	0.194
Other hospitalizations, median (IQR)	421.40 (3738.0)	53.6 (947.0)	1436.00 (8794.0)	280.5 (2631.3)	0.00 (3553.1)	0.0 (733.9)	

Abbreviations: IQR, interquartile range; PNH, paroxysmal nocturnal hemoglobinuria; PPPY, per patient per year; RBC, red blood cell; SD, standard deviation.

**Table 8:** Annual healthcare costs per patient per year for 38 patients treated with C5/3i during the period from the start to the end of treatment.

	From the Start to the End of Treatment (n = 37)
**A. Healthcare costs** (**EUR**)	**PPPY**
Total costs, mean (±SD)	302,120.9 (±66,912.5)
Total costs, median (IQR)	301,307.3 (56,595.2)
Hospitalizations, mean (±SD)	7420.8 (±31,077.5)
Hospitalizations, median (IQR)	54.0 (3255.8)
Drug prescriptions, mean (±SD)	293,712.2 (±54,017.8)
Drug prescriptions, median (IQR)	299,592.0 (55,133.5)
Specialist services, mean (±SD)	988.0 (±1226.5)
Specialist services, median (IQR)	590.0 (1451.3)
**B. Drug costs** (**EUR**)	**PPPY**
Total drug costs, mean (±SD)	293,712.2 (±54,017.8)
Total drug costs, median (IQR)	299,592.0 (55,133.5)
C5/3i, mean (±SD)	290,256.7 (±56,165.6)
C5/3i, median (IQR)	299,495.8 (62,292.9)
Other PNH-related drugs, mean (±SD)	1103.4 (±3202.8)
Other PNH-related drugs, median (IQR)	7.4 (104.4)
Other drugs, mean (±SD)	2352.1 (±9528.8)
Other drugs, median (IQR)	143.0 (522.4)
**C. Hospitalization costs** (**EUR**)	**PPPY**
Tot. hospitalization costs, mean (±SD)	7420.8 (±31,077.5)
Tot. hospitalization costs, median (IQR)	54.0 (3255.8)
PNH comorbidities, mean (±SD)	0.0 (±0.0)
PNH comorbidities, median (IQR)	0.0 (0.0)
PNH clinical manifestations, mean (±SD)	224.8 (±616.7)
PNH clinical manifest., median (IQR)	0.0 (0.0)
RBC transfusions, mean (±SD)	6333.5 (±31,204.9)
RBC transfusions, median (IQR)	0.0 (442.7)
Other hospitalizations, mean (±SD)	862.5 (±2123.7)
Other hospitalizations, median (IQR)	0.0 (364.8)

Abbreviations: IQR, interquartile range; PNH, paroxysmal nocturnal hemoglobinuria; PPPY, per patient per year; RBC, red blood cell; SD, standard deviation.

## Data Availability

All data used for the current study are available upon reasonable request from CliCon S.r.l. Società Benefit, which is the body that deals with data treatment and analysis in local health units.

## Appendix A

**Table A1:** List of comorbidities included in the Elixhauser Index with related ICD-9-CM codes.

Condition	ICD-9-CM Codes
Alcohol abuse	265.2, 291.1–291.3, 291.5–291.9, 303.3, 303.9, 305.0, 375.5, 425.5, 535.3, 571.0–571.3, 980, V11.3
Blood loss anemia	280
Cardiac arrhythmias	426.0, 426.10, 426.12, 426.13, 426.7, 426.9, 427–427.4, 427.6–427.9, 785.0, 996.01, 996.04, V5.0, V53.3
Chronic peptic ulcer disease (includes bleeding only if obstruction is also present)	531.7, 531.9, 532.7, 532.9, 533.7, 533.9, 534.7, 534.9
Chronic pulmonary disease	490–492, 493, 494–505, 506.4
Coagulation deficiency	286, 287.1, 287.3–287.5
Congestive heart failure	398.91, 402.01, 402.11, 402.91, 404.01, 404.03, 404.11, 404.13, 404.91, 404.93, 425.4–425.9, 428
Deficiency anemias	280.1–280.9, 281
Depression	296.2, 296.3, 296.5, 300.4, 309, 311
Diabetes with chronic complications	250.4–250.9, 775.1
Diabetes without chronic complications	250.00–250.3
Drug abuse	292.0, 304, 305.2–305.9, V65.42
Fluid and electrolyte disorders	253.6, 276
HIV/AIDS	042–044
Hypertension (without complications)	401.1
Hypertension (with complications)	402–405
Hypothyroidism	240.9, 434, 244, 246.1, 246.8
Liver disease	070.22, 070.23, 070.33, 070.44, 070.54, 070.6, 070.9, 456.0–456.2, 570, 571, 572.2-572.8, 573.3, 573.4, 573.8, 573.9, V42.7
Lymphoma	200–202, 203.0, 238.6
Metastatic cancer	196–199
Obesity	278
Other neurological disorders	330–331, 332.0, 333.4, 333.5, 334–335, 340, 341.1–341.9, 345, 347, 780.3, 784.3
Paralysis	334.1, 342, 343, 344.0–344.6, 344.9
Peripheral vascular disease	093.0, 437.3, 440, 441, 443.1–443.9, 447.1, 557.1, 557.9, V43.4
Psychoses	293.8, 295, 296.04, 296.14, 296.44, 296.54, 297, 298
Pulmonary circulation disorders	415.0, 415.1, 416, 417.0, 417.8, 417.9
Renal failure	403.01, 403.11, 403.91, 404.02, 404.03, 404.12, 404.13, 404.92, 404.93, 585, 586, 588, V42.0, V45.1, V56
Rheumatoid arthritis/collagen vascular diseases	446, 701.0, 710.0–710.4, 710.8, 710.9, 711.2, 714, 719.3, 720, 725, 728.5, 728.89, 729.30
Solid tumor without metastasis	140–172, 174–195
Valvular disease	093.2, 394–397, 397.9, 424, 746.3–746.6, V42.2, V43.3
Weight loss	260–263, 783.2, 799.4

**Table A2:** List of codes of PNH-related comorbidities, clinical manifestations and in-hospital procedures with related codes.

Condition	ICD-9-CM Codes
**PNH-related comorbidities**	
AA and other BMF syndromes	284.x
MDS	238.72, 238.75
Non-hematological malignancy	140.X, 199.X
Hematological malignancy	200.X, 208X
Acute myelogenous leukemia	205.00
Myeloproliferative disorder	238.76
Myelofibrosis	289.83
**Clinical manifestations of PNH**	
Anemia (excluding AA)	280.x–282.x, 283.0x, 283.1x, 283.9x, 285.x
Respiratory infections	460–466, 472, 473, 476, 480-488
Infections	001–139
Chronic kidney disease	403.x, 404.x, 585.x
Sepsis	038, 790.7, 995.91, 995.92
Abdominal pain	789.0x, 789.6
Dysphagia	787.2x
Dyspnea	786.02, 786.05
Erectile dysfunction	607.84
Fatigue	780.71, 780.79
Kidney and urinary tract infections	590.0, 590.1, 590.80, 590.9, 595, 597.80, 599.0
Pulmonary hypertension	416.0, 416.8
Major adverse vascular events	
Myocardial infarction (not STEMI)	410.71
Transient ischemic attack	435.x
Unstable angina	411.1
Gangrene (nontraumatic, nondiabetic)	785.4
Cerebral arterial occlusion/CVA	434.01
Thrombosis	
Phlebitis and thrombophlebitis of intracranial venous sinuses	325.x
Pulmonary embolism and infarction	415.1
Occlusion of cerebral arteries	434
Arterial embolism and thrombosis of lower extremity	444.22
Phlebitis and thrombophlebitis of femoral vein (deep) (superficial)	451.11
Phlebitis and thrombophlebitis of deep veins of lower extremities, other	451.19
Portal vein thrombosis	452
Other venous embolism and thrombosis	453
Vascular insufficiency of intestine	557
Thrombophlebitis/deep vein thrombosis	451.x, 453.4x, 453.5x, 453.72, 453.82
Pulmonary embolus	415.1x
Renal vein thrombosis	453.3
Acute peripheral vascular occlusion	443.9
Vein thrombosis	452, 453.x
Arterial thrombosis	444.x
Cerebral venous occlusion	434.x
Renal arterial thrombosis	593.81
Phlebitis and thrombophlebitis of intracranial venous sinuses	325.x
Pulmonary embolism and infarction	415.1
Occlusion of cerebral arteries	434
**Other conditions or procedures**	**Codes** (**ICD-9-CM, exemption, procedure**)
PNH	ICD-9-CM code 283.2; exemption code RD0020
RBC transfusion	ICD 9 diagnosis code V582; procedure 99.0
Bone marrow transplant	ICD-9-CM code 284; procedure 41.0

## Appendix B

Epidemiological analysis of the overall PNH population (pediatric and adult subjects).
